# Flow-Cytometric Phosphoprotein Analysis Reveals Agonist and Temporal Differences in Responses of Murine Hematopoietic Stem/Progenitor Cells

**DOI:** 10.1371/journal.pone.0003776

**Published:** 2008-11-20

**Authors:** Demetrios Kalaitzidis, Benjamin G. Neel

**Affiliations:** Hematology/Oncology-Cancer Biology Program, Beth Israel Deaconess Medical Center, Harvard Medical School, Boston, Massachusetts, United States of America; Oklahoma Medical Research Foundation, United States of America

## Abstract

Hematopoietic stem cells (HSCs) are probably the best-studied adult tissue-restricted stem cells. Although methods for flow cytometric detection of phosphoproteins in hematopoeitic progenitors and mature cells are available, analogous protocols for HSC are lacking. We present a robust method to study intracellular signaling in immunophenotypically-defined murine HSC/progenitor cell (HPC)-enriched populations. Using this method, we uncover differences in the response dynamics of several phosphoproteins representative of the Ras/MAP-Kinase(K), PI3K, mTOR and Jak/STAT pathways in HSC/HPCs stimulated by Scf, Thpo, as well as several other important HSC/HPC agonists.

## Introduction

HSCs self-renew and give rise to all hematopoietic cells. Adult murine HSCs can be prospectively enriched by their low expression of multiple lineage markers (LIN^−^) including CD127, and high expression of c-Kit (hereafter, Kit) and Sca-1, as well as several other markers [1]. HSC with long-term (LT) repopulating activity (CD34^−^) can be further purified from short-term (ST)-HSC and multipotent progenitors (MPP) (CD34^+^) using CD34 antibodies. MPPs give rise to lineage-restricted progenitors with minimal self-renewal and more restricted differentiation capacities, including common lymphoid progenitors (CLPs; LIN^−^c-Kit^lo^Sca-1^lo^CD34^+^CD127^+^), lymphoid-primed MPPs (LMPPs; LIN^−^c-Kit^+^Sca-1^+^CD34^+^Flt3^+^CD127^+/−^), and common myeloid progenitors (CMPs) [1]. CMPs (LIN^−^c-Kit^+^Sca-1^−^CD34^+^FcγRII/III^lo^CD127^−^) yield granulocyte/monocyte progenitors (GMPs: LIN^−^c-Kit^+^Sca-1^−^CD34^+^FcγRII/III^+^CD127^−^) and megakaryocyte/erythroid progenitors (MEPs:LIN^−^c-Kit^+^Sca-1^−^CD34^−^FcγRII/III^−^CD127^−^), which ultimately give rise to all red and white blood cells, including platelets. The LIN^−^c-Kit^+^Sca-1^+^ (LSK) fraction of the bone marrow (BM) is enriched for HSCs and MPPs, whereas the LIN^−^c-Kit^+^Sca-1^−^ (LK) fraction contains CMPs, GMPs and MEPs [1].

Much progress has been made in determining the physiological function of HSCs/HPCs. Less is known about their biochemical responses to various agonists, largely because traditional approaches (e.g., immunoblotting) are not applicable to such rare cells. Recently, murine HSC purified by Fluorescence-Activated Cell Sorting (FACS) were stimulated *ex vivo*, and changes in their phosphoprotein levels were visualized by immunofluoresence [2]. Although this approach provides some insights into HSC signaling and also permits the analysis of protein subcellular localization, it only allows analysis of one cellular population at a time and is subject to the limitations of immunofluorescence (e.g., difficulties in quantification, photobleaching, etc.). BM cells also have been stimulated *ex vivo*, then fixed, permeabilized, immunostained for surface and intracellular markers, and analyzed by FACS [3]. However, this method only permits analysis of the highly heterogeneous LIN^−^Kit^+^ population. There is one report of intracellular staining of LIN^−^ cells that were first surface-stained for Sca-1 and Kit, and then fixed and saponin-permeabilized with a commercial kit [4]. However, this permeabilization agent may not be optimal for HSC/HPC signaling studies (see below).

We have optimized conditions to analyze signal transduction pathways in phenotypically-defined HSC/HPC subsets. We focused on two well-characterized agonists, stem cell factor (Scf) and thrombopoietin (Thpo), as well as several other important HSC/HPC growth factors and cytokines, and demonstrate robust responses in several well-studied signaling pathways. Using our approach we observed signaling cross-talk between the MEK and mammalian target of rapamycin (mTOR) pathways, converging on ribosomal protein S6 phosphorylation in HSC/HPC.

## Results

### Fixation conditions for retaining surface antigenicity of sorted Lin^−^ cells

**Figure 1:**
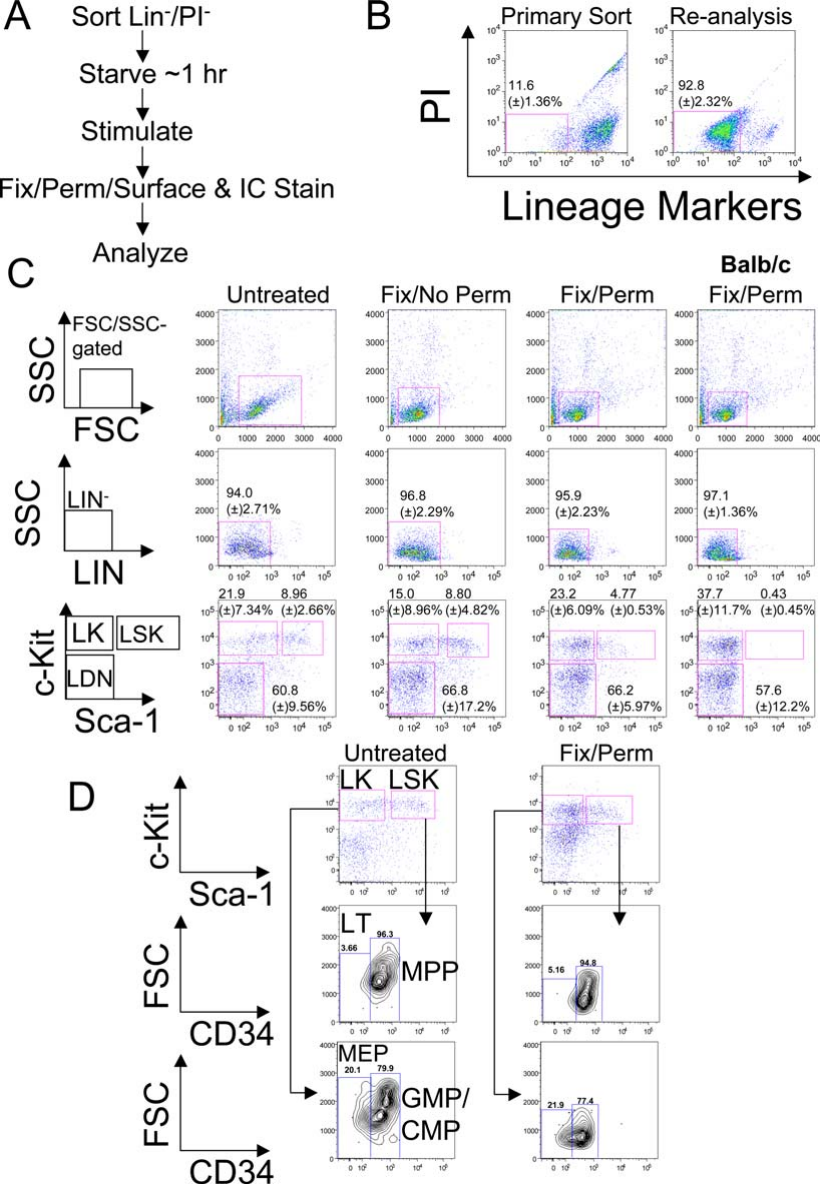
Surface marker expression in paraformaldedyde (PFA)-fixed (Fix), acetone-permeabilized (Perm) LIN^−^/PI^−^ cells. A, Schematic showing approach for enriching HSC/HPC populations and analyzing intracellular (IC) and extracellular (surface) antigens. B, Murine BM cells were stained with LIN markers and PI. “Primary Sort” indicates typical gates for LIN^−^/PI^−^ cells after debris and cell clumps were gated using forward scatter/side scatter (FSC/SSC). “Re-analysis” represents cells analyzed for LIN/PI staining immediately after sorting. Numbers indicate percentage (±) standard deviation (SD) (n = 5) of LIN/PI-stained BM cells before and after sorting. C, Cells were sorted as in (B), incubated *in vitro* at 37°C for ∼1 hour in 2% FBS/IMDM, and either left untreated, fixed with 1.5% PFA without permeabilization, or fixed with PFA and subsequently permeabilized with ice-cold ∼100% acetone. Cells were then stained with Kit (APC-conjugate) and Sca-1 (PE-conjugate) antibodies and analyzed with a flow cytometer. Shown are pseudocolor dot plots and gating schemes, along with staining of BM from BALB/c mice, which express low levels of Sca-1 [9]. The percentage of the parental gate ±SD from 4 independent experiments is indicated. LK, LIN^−^Kit^+^Sca-1^−^, LSK, LIN^−^Kit^+^,Sca-1^+^, LDN, LIN^−^Kit^−^Sca-1^−^. A minimum of 2,500 events was collected (not counting cells with high SSC and low FSC, gated as in panel (B), and hereafter, termed “FSC/SSC-gated events”). D, LIN^−^/PI^−^ cells were left untreated or fixed, permeabilized, and stained for Sca-1 and Kit (as above), as well as CD34 (Pacific Blue-conjugate). Shown are pseudocolor dot plots, contour plots, and the percentage of the parental gates (from one of several experiments). A minimum of 1,500 FSC/SSC-gated events was collected. CD34 gates were set according to fluorescence minus one controls (data not shown). LT, long-term HSC, MPP, multipotent progenitors, GMP, granulocyte/monocyte progenitors, CMP, common-myeloid progenitors, MEP, megakaryocyte/erythroid progenitors.

We sought a robust protocol for multi-parametric, quantitative analysis of intracellular signaling in phenotypically-defined murine stem/progenitor cell populations [5]–[7]. Previous studies showed that many extracellular proteins maintain sufficient antigenicity for FACS after paraformaldehyde (PFA) fixation and alcohol-based permeabilizaton [5], and established that alcohol- (or ketone-) based permeabilization is superior to detergents for preserving intracellular phosphoproteins [6]. A major barrier to applying these methods to HSCs/HPCs arises from the requirement that multiple lineage markers be used to enrich for the LIN^−^ HSC/HPC compartment (Materials and Methods). Some important extracellular antigens lose antigenicity after fixation/permeabilization [5], and contamination of the LIN^−^ compartment with LIN^+^ cells rendered artifactually “LIN^−^” by fixation/permeabilization could seriously confound analysis. We decided to enrich for HSC/HPC by first removing LIN^+^ and dead cells (propidium iodide [PI]^+^) by FACS (Figure 1A and B). We avoided positive selection with HSC/HPC-specific antigens to limit potential antibody-triggered signaling events [8]. LIN^−^ cells represent a minority of murine BM (<15%), and we obtained this population at reasonably high purity with an average yield of 5.75×10^5^ (±) 2.38×10^5^ cells/mouse (mean±standard deviation [SD]; n = 13 independent experiments; Figure 1B). A minimum of 1×10^5^ LIN^−^PI^−^ cells, comprising ∼100 LSK and >200 LK cells after fixation/permeabilization, is sufficient for analysis of signaling events following brief *ex vivo* culture and agonist stimulation (Figure 1A and data not shown).

We next sought conditions that preserve surface antigens on HSC/HPC. Consistent with previous reports [3], [5], Kit staining was maintained after PFA fixation and permeabilization with multiple agents (Figure 1C and S1). However, Sca-1 antigenicity was destroyed after PFA fixation and methanol permeabilization [3], and significantly reduced following PFA fixation and permabilization with either ethanol, methanol, isopropranol, Triton (0.50%), or two concentrations of saponin. Saponin permeabilization also increased non-specific binding (Figure S1). There was considerable retention of Sca-1 antigenicity on LIN^−^Kit^+^ cells following acetone or 0.10% Triton treatment (Figure 1C and S1), although the median fluorescent intensity (MFI) of these cells relative to untreated or PFA-fixed cells was reduced. After adjusting the gating to account for the reduced MFI, a distinct population of LSK cells could still be identified. Acetone was superior to Triton in preserving Sca-1 antigenicity (as indicated by the significantly higher MFI) in the LIN^−^Kit^+^ population (Figure S1), and was reported previously to provide superior preservation of intracellular phosphoprotein epitopes (compared to detergents) [6]; thus, we used acetone in all subsequent experiments.

Sca-1 staining on LIN^−^Kit^+^ cells was specific, as BM from BALB/c mice, which express low/no levels of Sca-1 [9], showed a substantially reduced LSK population compared to the C57BL/6 BM used above and in all other experiments (Figure 1C). The percentage of cells retaining CD34 antigenicity (and the CD34 MFI) also was comparable in untreated and PFA-fixed/acetone-permeabilized cells (Figure 1D), allowing discrimination of LT-HSC (CD34^−^) from ST-HSC/MPP (CD34^+^) [10] and GMP/CMP (CD34^+^) from MEP (CD34^−^)-enriched populations within the LK compartment [11]. We also obtained satisfactory staining for fms-like tyrosine kinase 3/fetal liver kinase 2 (Flt3/Flk2) and CD48, either of which can further be used to discriminate between HPCs, MPPs and LT/ST-HSC [12]–[14] (Figure S2, Materials and Methods S1). However, we could not obtain conditions for FcγRII/III (PE-Cy7-conjugate of clone 93) staining, which would allow discrimination of GMPs from CMPs, nor for CD150/Slam (with either PE or PE-Cy7 conjugates of clone TC15-12F12.2), which, like CD34 or Flk2/Flt3, also can discriminate LT-HSC from MPPs (data not shown).

### Detection of agonist-evoked changes in intracellular phosphoproteins in HSC/HPC

**Figure 2:**
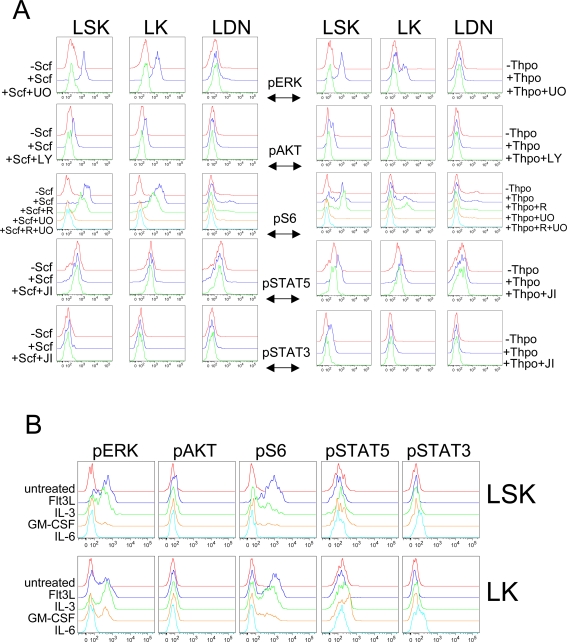
Differential responses of defined LIN^−^ populations to various agonists. A, LIN^−^/PI^−^ BM cells were sorted and incubated in vitro in 2% FBS/IMDM for ∼1 hour. Where indicated, cells were pre-incubated with inhibitors for 30 minutes–1 hour prior to stimulation for 5 minutes with the indicated agonists (100 ng/ml Scf or 50 ng/ml Thpo). Cells were fixed immediately, permeabilized, washed several times and stained for extracellular antigens (as in Figure 1) and the indicated phosphoproteins. Data are presented as the log of fluorescence intensity on the x-axis and the % maximal value on the y-axis, and are representative of at least 2–3 independent experiments (See Figure S3 for quantification of the changes in MFI in response to the various treatments). Minima of 3,700 and 1,300 FSC/SSC-gated events were collected for the Scf and Thpo experiments, respectively. UO: 10 µM UO126 for 30 minutes, LY: 20 µM Ly294002 for 30 minutes, R: 100 nM rapamycin for 30 minutes, JI,:1 µM Jak inhibitor I for 1 hour. (See text and Materials and Methods for the specific phosphorylation sites interrogated on each phosphoprotein). B, Cells were treated with the indicated agonists (50 ng/ml Flt3L, 10 ng/ml IL-3, 20 ng/ml Gm-csf, 20 ng/ml IL-6) for 5 minutes and processed as in (A). Data are displayed as in (A) and are representative of at least 2 independent experiments. A minimum of 2,700 FSC/SSC-gated events was collected (See Figure S5 for quantification of the responses to the various treatments).

We stimulated LSK, LK and LIN^−^Kit^−^Sca-1^−^ (LDN) cells with two agonists that have well-established roles in HSC/HPC physiology, Scf or Thpo, and asked if intracellular phosphoproteins could be detected. The receptor for Scf, Kit, is expressed on all HSC/HPC (see above), and the Thpo receptor, c-Mpl, is expressed (at the mRNA level) in HSC/CMP/MEP but not GMP [11]. Sorted LIN^−^/PI^−^ cells were cultured briefly in low serum-containing media for ∼1 hour (Figure 1A, Materials and Methods), stimulated for 5 min with Scf (100 ng/ml) or Thpo (50 ng/ml), fixed and permeabilized as above, stained simultaneously for surface and intracellular antigens, and analyzed by flow cytometry (Figure 2A). As representatives of major cytokine and growth factor signaling pathways, we probed for phosphorylated(p)-ERK1/2 (Thr202/Tyr204), p-AKT (Ser473), p-ribosomal protein S6 (Ser235/236), p-STAT5 (Tyr694), and p-STAT3 (Tyr705).

Following Scf treatment, robust pERK and pS6 responses were observed in LSK and LK, but not LDN cells (Figure 2A; quantified in Figure S3). The pERK response was specific, as it was eliminated by pre-treating cells with the MEK inhibitor UO126 (Figure 2A). S6 can be phosphorylated on Ser235/236 as a consequence of mTOR or ERK activation [15]. Scf-evoked phosphorylation of these residues at 5 min in either LSK or LK cells was primarily UO126-, not rapamycin-sensitive, and therefore MEK, rather than mTOR-dependent (Figure 2A). We also observed a small, PI3K-dependent response, in pAKT (Figure 2A). We failed to detect significant Scf-evoked increases in STAT3 or STAT5 phosphorylation in any of the cell populations examined (Figure 2A). Although this could indicate a lower sensitivity for detecting pSTAT than other p-epitopes, phosphorylation of both STAT proteins was detected in response to Thpo, as well as several other agonists (see below).

Thpo also induced a strong increase in pERK and pS6 in LSK cells. Unlike Scf, which affected LSK and LK populations equally, Thpo-evoked responses were lower in LK cells (Figure 2A). Thpo also evoked a small, but specific, increase in pAKT (Figure 2A). Unlike Scf, Thpo induced significant increases in pSTAT5 and pSTAT3 in LSK cells and in pSTAT5 alone in LK cells (Figure 2A). Consistent with our findings, Thpo-evoked increases in pAKT, pSTAT5 and pSTAT3 were observed previously in LSK CD34^−^ cells using immunofluorescence-based assays [2], [16]. Treatment with either Scf or Thpo and/or various kinase inhibitors had no effect on surface marker antigenicity in either cell population (Figure S4).

We also assessed phosphoprotein responses to other agonists commonly used *ex vivo* in HSC/HPC assays and culture conditions. Flt3 ligand (Flt3L) evoked strong increases in pERK and pS6 in most LSK cells and a minority of LK cells, and a smaller response in pAKT levels in LSK cells (Figure 2B; quantified in Figure S5). Like Scf, also a receptor tyrosine kinase agonist, Flt3L failed to evoke detectable changes in STAT phosphorylation (Figure 2B). Interleukin-3 (IL-3) elicited stronger responses in LK cells than in LSK cells, and increased pERK, pS6, and to a lesser extent pSTAT5 (Figure 2B and S5). Granulocyte/macrophage colony-stimulating factor (Gm-csf) evoked small increases in pERK, pS6 and pSTAT5, predominantly in LK cells, consistent with Gm-csf receptor levels being detected in GMP [17] (Figure 2B and S5). In both LSK and LK cells, IL-6 only evoked STAT3 phosphorylation with no changes in any of the other phosphoproteins analyzed (Figure 2B and S5).

**Figure 3:**
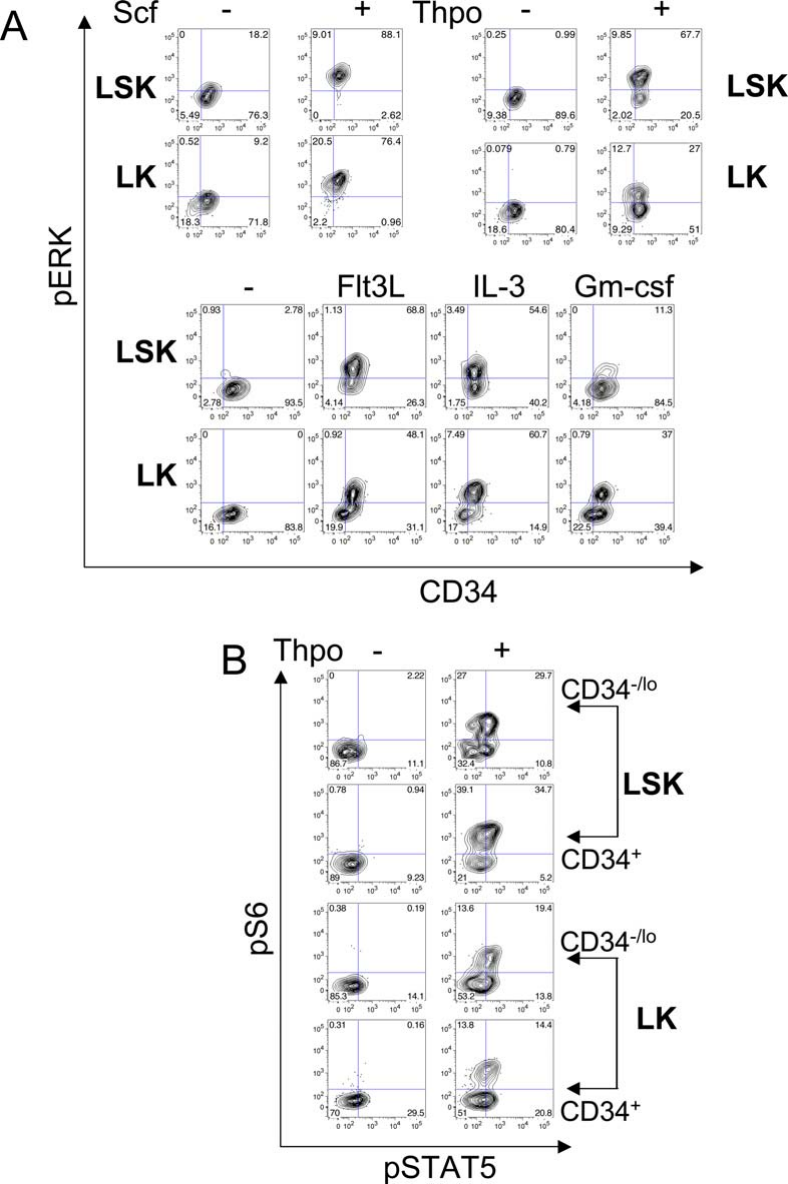
Detection of phosphoproteins in CD34-defined LSK/LK subsets. A, Cells were treated as in Figure 2A and gated for CD34 expression and pERK (Thr202/Tyr204) levels. Contour plots and percentage of parental gates are shown from a representative experiment performed at least twice. Minima of 6,000, 4,700, and 2,700 FSC/SSC-gated events were collected for the Scf, Thpo, and other agonist experiments, respectively. Using CD34-gated HSC/HPC subsets permit analysis of signaling events in more discretely defined HSC/HPC populations. B, Cells were treated with Thpo for 5 minutes, processed as described above, and stained simultaneously for surface markers, pS6 (Ser235/236), and pSTAT5 (Tyr694) in the indicated LSK and LK subsets (gated as in Figure 1D). Data are representative of 2 independent experiments, with a minimum of 7,400 FSC/SSC-gated events collected. Note the ability to simultaneously detect multiple phospho-epitopes in defined HSC/HPC subsets.

Using CD34 staining, we also analyzed signaling responses in more refined LSK/LK subsets (Figure 3, S6, and S7). Scf evoked nearly uniform ERK phosphorylation in LSK and LK subsets. In contrast, Thpo responses were more heterogeneous. Most LSK cells increased pERK levels in response to Thpo, yet a substantial minority of CD34^+^/CD34^−^ LSK cells did not respond (Figure 3A). The response to Thpo in LK cells was also heterogeneous, with only minor fractions of CD34-positive and -negative cells showing activation (Figure 3A). These findings are consistent with expression of c-Mpl on CD34^+^ CMPs and CD34^−^ MEPs [11]. Flt3L also evoked a heterogeneous response in LSK and LK cells. In both, LSK and LK cells, elevated Flt3L-evoked pERK levels were mostly detected in CD34^+^ cells, although some of these cells showed no increase in pERK (Figure 3A and S2). IL-3-evoked phosphorylation of ERK was equivalent in CD34^+^ and CD34^−^ LSK cells, but was stronger in CD34^+^ LK cells (as opposed to CD34^−^ cells). In contrast, Gm-csf responses were more apparent in LK cells, with a subset of CD34^+^ LK cells responding (Figure 3A) [17]. Other phosphoprotein signaling responses measured in LSK/LK CD34 subsets are presented in Figure S6 and S7. Finally, by using antibodies to pS6 and pSTAT5, we found that several phosphoproteins can be quantified simultaneously in single cells from all of the LSK and LK CD34 subsets tested (Figure 3B).

### Kinetics of signaling events in HSC/HPC

**Figure 4:**
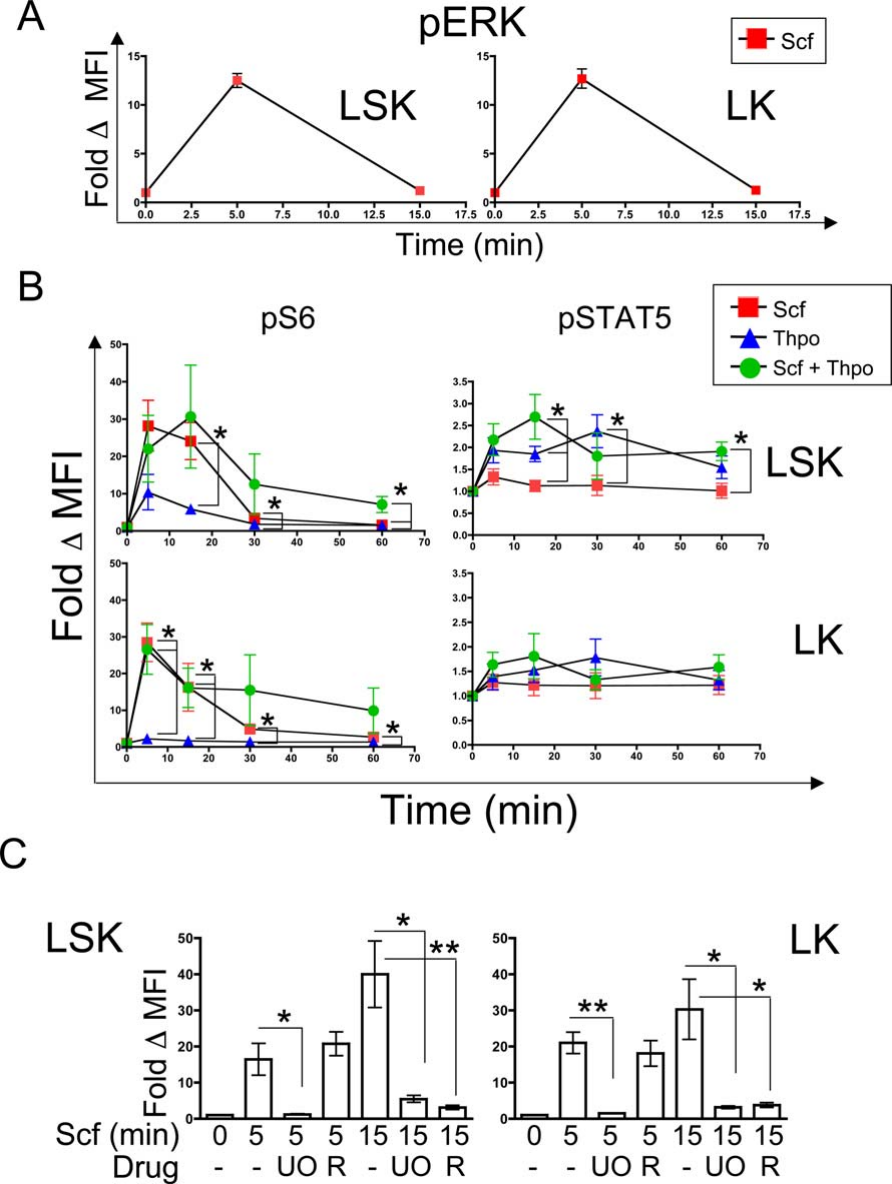
Kinetic and pathway analysis in HSC/HPC subsets. A, Cells were treated with Scf for the indicated time in minutes (min) and processed as above. Levels of pERK were monitored and normalized to untreated controls in the indicated populations (Fold Δ MFI). Data represent the mean of two independent experiments ±SD. B, Cells were treated for various times (min) with the indicated agonists and levels of pS6 (Ser235/236) and pSTAT5 (Tyr694) in the HSC/HPC subsets were quantified. Data are normalized to untreated controls in each indicated population (Fold Δ MFI). Data points represent the average from 4 independent experiments (except for the Scf+Thpo 30 minute time point, which is from 3 experiments) shown ±SEM. (*P<.05). Red squares, Scf, blue triangles, Thpo, green circles, Scf+Thpo. C, Cells were treated with Scf and the indicated inhibitors (Drug) over the depicted amount of time (min) and pS6 (Ser235/236) levels were monitored in LSK and LK cells. Levels of pS6 were quantified as above, and the means of at least 4 independent experiments ±SEM are shown. (*P<.05, **P<.01). UO, UO126, R, Rapamycin.

Our initial analyses were performed to assess the selectivity and sensitivity of our protocol and focused on an early time point (5 minutes), which would presumably reveal strong responses and be less influenced by pathway cross-talk. Subsequently, we characterized later responses in several phosphoproteins. Scf-evoked ERK phosphorylation (Thr202/Tyr204) was elevated 5 minutes post-stimulation, rapidly declined by 15 minutes and remained at baseline levels for at least 30 minutes post-stimulation in LSK and LK cells (Figure 4A and data not shown). We observed similar kinetics of response in pERK in response to Thpo (data not shown). Scf- and Thpo- evoked S6 phosphorylation typically peaked by 5 minutes post-stimulation in both LSK and LK cells, declined gradually starting at 15 minutes, and returned to near-baseline levels at 30 minutes for Thpo and 1 hour for Scf (Figure 4B). Scf-evoked pS6 responses were significantly higher than Thpo-evoked responses in LSK and LK cells throughout the time course (Figure 4B). The combination of Scf and Thpo did not lead to an enhanced pS6 response relative to Scf treatment at most time points in either LSK or LK cells (Figure 4B). However, S6 phosphorylation was sustained significantly in LSK, but not LK cells, when both agonists were used compared to either agonist alone (Figure 4B; Fold change in MFI at 60 minutes with Scf+Thpo: 7.07±2.14; Scf alone: 1.62±0.13 p = 0.04; Thpo alone: 1.47±0.15, p = 0.04).

As noted above (Figure 2 and S3), pSTAT5 levels were higher in Thpo-stimulated LSK cells, compared to Scf-stimulated cells. These levels appeared to peak at 15 minutes post-Thpo stimulation, and remained elevated slightly even at 60 minutes in LSK cells (Figure 4B). Scf did not affect STAT5 phosphorylation appreciably over this time course either alone or in combination with Thpo treatment (Figure 4B).

### Differential control of S6 phosphorylation at Ser235/236 in HSC/HPC

Scf-evoked phosphorylation of ribosomal protein S6 at Ser235/236 at 5 minutes was completely sensitive to MEK inhibition and insensitive to mTOR inhibition (Figure 2A and S3). However, S6 phosphorylation remained elevated at 15 minutes post-Scf addition, whereas pERK levels declined rapidly after 5 minutes (Figure 4A and B). At 15 minutes post-Scf addition, pS6 levels were both MEK- and mTOR-dependent in LSK and LK cells, as the fold change in MFI for pS6 was reduced by either UO126 or rapamycin pre-treatment. These data are similar to previously reported results on mouse and human cell lines [15], [18], and provide proof of principle for the utility in pathway analysis of our phosphoflow protocol in HSC/HPC-enriched populations.

## Discussion

We have developed a protocol that utilizes the multi-parametric and quantitative attributes of FACS to examine multiple phosphorylation events at the single cell level in highly purified HSC/HPC populations. Previous studies have only been able to analyze signaling events in bulk hematopoietic progenitors by flow cytometry [3] or one cellular parameter at a time by immunofluorescence-based techniques [2]. Although still limited by low target cell numbers and loss of a significant proportion of the starting material post fixation/permeabilization and washing steps, our protocol permits the simultaneous analysis of multiple cellular (HSC, MPP, GMP/CMP, MEP), and intracellular parameters in HSC/HPC, akin to studies of more mature hematopoietic cells [6].

Intracellular signaling networks in HSC/HPC have remained largely unexplored territory, mostly due to the rarity of these cell populations. Here we have analyzed signal transduction responses to several HSC/HPC agonists, including Scf and Thpo, both of which are important *in vivo* for HSC function and can promote survival and enhance the proliferation of HSC/HPC *ex vivo* [2], [19]–[23]. Although Scf elicited rapid responses in some intracellular pathways that we interrogated (Ras/MAPK, PI3K/AKT) in all of the HSC/HPC cellular subsets we analyzed, Thpo stimulation profiles were stronger in HSC/MPP (compared to GMP/CMP or MEP) and consisted of the activation of a broader array of intracellular pathways (Ras/MAPK, PI3K/AKT, JAK/STAT). Other agonists, such as Flt3L, appeared to elicit stronger responses in LSK compared to LK cells, whereas Gm-csf elicited stronger responses in LK cells. These differences in response could reflect a heterogeneous expression of receptors in LSK and LK cells, as well as differences in the intrinsic sensitivity to these agonists between populations. We detected a significant synergistic effect of Scf and Thpo relative to either treatment alone on pS6 levels in LSK, resulting in a sustained pS6 response (Figure 4B). It is tempting to speculate that the reason for biological synergy (in terms of colony formation or proliferation/survival of HSC/HPC) previously observed between Scf and Thpo [2], [19]–[21] reflects sustained Ras/MAPK and mTOR signaling, although further genetic- or pharmacology-based biological assays are required to explore this point. Recent studies have demonstrated that both Thpo and Kit are required to maintain the steady-state pool of adult quiescent HSC [22], [24]. While STAT5 plays a significant role in HSC function [16], [25], [26], the role of members of the Ras/MAPK or mTOR pathway in HSC biology remains largely unexplored. Our data suggest that these pathways likely play a role downstream of agonist stimulation in HSC/HPC.

We also simultaneously assessed responses in pS6/pSTAT5 in CD34-LSK and LK subsets (Figure 3B). In all populations analyzed, approximately half of the cells respond to Thpo stimulation by elevating both pS6 and pSTAT5, but the rest only elevate pS6 (Figure 3B). This could reflect the relative hypersensitivity of the pathways downstream of Thpo leading to S6 phosphorylation over signaling to STAT5, lower sensitivity of our conditions for detecting pSTAT5, or intrinsic heterogeneity in the cell types present in these compartments. Regardless, the physiological relevance of this heterogeneity in signaling responses merits further investigation.

Our approach can be used along with selective inhibitors to examine pathway-dependencies. For example, our analysis of Scf-evoked S6 phosphorylation in primary HSC/HPC is largely in agreement with that of Roux et al. [15], who demonstrated that serum-evoked S6 phosphorylation at Ser235/236 in human cell lines (HEK 293) is dependent on an early MEK-dependent pathway (through RSK) and a later MEK- and mTOR-dependent pathway. Our data are also consistent with the Mek-inhibitor sensitivity of Ser235/236 phospohorylation in mouse embryonic fibroblasts doubly deficient for the mTOR downstream kinases S6K1/2 [18]. Our results suggest a model in which Scf stimulation leads to ERK activation that likely leads to RSK1/2 activation and S6 phosphorylation at Ser235/236 at 5 minutes post stimulation. At 15 minutes, S6 phosphorylation, is still partially UO126- and rapamycin- sensitive, although levels of pERK have returned to baseline at this time point. ERK activation also can lead to TSC2 phosphorylation and inactivation [27]. Our data suggests the intriguing possibility that ERK mediated TSC2 inactivation is required along with mTOR-dependent phosphorylation of S6 to sustain high levels of pS6 in HSC/HPC. Direct testing of this model will be possible with the availability of appropriate phosphospecific antibodies.

Finally, our protocol should be amenable to the study of signal transduction abnormalities in leukemia stem cells (LSC), as these cells often share phenotypic similarities with normal HSC/HPC [17], [28]. The effects of drugs that may affect deregulated signal transduction pathways in leukemia can be readily tested in cell populations expressing surface epitopes examined in this study.

## Materials and Methods

### Antibodies, reagents

Lineage-specific antigens were stained with the following antibodies, all conjugated to PE-Cy5: CD3ε (145-2C11, BioLegend, San Diego, CA), CD4 (RM4-5, eBioscience, San Diego, CA), CD8α (53-6.7, BioLegend), CD19 (6D5, BioLegend), CD45R (RA3-6B2, eBioscience), CD127 (A7R34, eBioscience), Ly-6G (RB6-8C5, eBioscience), and Ter119 (TER119, BioLegend). Antibodies against c-Kit (APC-conjugated, 2B8, eBioscience), Sca-1 (PE-conjugated, D7, eBioscience) and CD34 (Pacific Blue conjugated, RAM34, eBioscience) were also used. The following monoclonal antibodies were used to detect intracellular phosphoproteins: pERK [Thr202/Tyr204] (Alexa Fluor [Ax] 488-conjugated, E10, Cell Signaling, Danvers, MA), pAKT [Ser 473] (Ax488-conjugated, 193H12, Cell Signaling), pS6 [Ser235/236] (Ax488-conjugated, D57.2.2E, Cell Signaling), pSTAT5 [Tyr694] (PE-Cy7- conjugated, 47, Becton Dickinson [BD] Biosciences, San Jose, CA), and pSTAT3 [Tyr705] (Ax488-conjugated, D3A7, Cell Signaling). UO126, Ly294002, and Jak Inhibitor I were purchased from Calbiochem (La Jolla, CA). Rapamycin was purchased from Cell Signaling. Recombinant murine forms of Scf, Thpo, Flt3L, IL-3, Gm-csf and IL-6 were purchased from Peprotech (Rocky Hill, NJ) and dissolved in 0.10% BSA/PBS. PFA was purchased from Electron Microscopy Sciences (Hatfield, PA).

### Cell preparation and FACS

BM was harvested from both limbs of 8–12-week-old C57BL/6 or BALB/c mice (Charles River Laboratories, Wilmington, MA) euthanized by CO_2_ asphyxiation, followed by cervical dislocation, flushed into cold 2% FBS/IMDM and passed through a 40 µm cell strainer (BD Falcon). Cells were collected by centrifugation, and red blood cells were lysed in 0.16 M NH_4_Cl on ice. Cells were then washed, centrifuged, resuspended in 400 µl of cold 2% FBS/IMDM, and stained with saturating levels of antibodies against the above lineage markers for ∼15 minutes on ice. After washing, cells were passed through a 40 µm cell strainer into 5-ml polypropylene round-bottom tubes (BD), and stained with 1 µg/ml PI (Invitrogen, Carlsbad, CA). LIN^−^/PI^−^ cells were purified using a FACSAria (BD). All mouse studies were approved by our Institutional Animal Care and Use Committee.

### Cell treatment and staining

Cells were fixed, permeabilized, and stained essentially as described previously, but using PFA fixation, followed by permeabilization with acetone [5], [6]. Sorted cells were placed into Eppendorf tubes, pelleted at 6,000 rpm on a benchtop centrifuge, and resuspended in 2% FBS/IMDM. A minimum of 1×10^5^ cells was aliquoted into Eppendorf tubes. After fixation, permeabilizeation, staining and several washes, there is considerable cell loss. Staining the above minimum of cells ensures that adequate events are acquired during re-analysis for statistically meaningful results (e.g., ∼100 LSK cells, >200–300 LK cells). Where indicated, cells were treated with various drugs and/or agonists (5 minutes with 100 ng/ml Scf, 50 ng/ml Thpo, 50 ng/ml Flt3L, 10 ng/ml IL-3, 20 ng/ml Gm-csf, or 20 ng/ml IL-6) at 37°C in a humidified incubator, and fixed immediately after the desired treatment times in 1.5% paraformaldehyde (final concentration) for 10 minutes at room temperature. Untreated (minus) cells were received 0.10% BSA/PBS and vehicle (DMSO). Fixed cells were collected by centrifugation at 6,000 rpm for 3 minutes, and resuspended in ∼100% ice-cold acetone (Fisher Scientific, Pittsburgh, PA), added drop-wise with simultaneous gentle vortexing. After ∼10 minutes on ice, cells were collected as above, washed twice with 1 ml of PBS/0.50%BSA/0.02%NaN_3_, and aliquoted into the number of Eppendorf tubes desired for intracellular stainings (100 µl each). Cells were stained for 20 minutes at room temperature in the dark with the indicated antibodies at the following final concentrations: anti-Sca-1 (2 ng/µl), anti-Kit (4 ng/µl), anti-CD34 (2 ng/µl for fix/perm cells; 5 ng/µl for untreated cells), anti-pERK (2 ng/µl), anti-pAKT (0.5 ng/µl), anti-pS6 (0.1 ng/µl), anti-pSTAT5 (1∶5 or 1∶10; no concentration specified by supplier), and anti-pSTAT3 (4 ng/µl). Cells were then washed with 10 volumes of PBS/0.50%BSA/0.02%NaN_3_, placed in 5-ml polystyrene round-bottom tubes (BD) in 300 µL of PBS/0.50%BSA/0.02%NaN_3_, and analyzed on an LSRII (BD). Compensation was calculated by using whole BM cells stained with directly-conjugated anti-CD45R antibodies (FITC, PE, PE-Cy5, APC, PE-Cy7, Pacific Blue). Data were analyzed using FlowJo software (TreeStar, Ashland, OR).

### Statistical analysis

The statistical significance of differences between population means was assessed by 2-tailed unpaired Student *t* test.

## Supporting Information

Materials and Methods S1 (0.03 MB DOC)

Figure S1 Effect of fixation/permeabilization conditions on Sca-1 staining. A, Cells were either left untreated or fixed with PFA without permeabilization, and subsequently stained for Kit and Sca-1. B, Cells were treated as in (A), and permeabilized with the indicated agents post-fixation. A mimimum of 1,400 FSC/SSC-gated events was collected for each treatment. Saponin was included in the permeabilization and wash/staining buffer when used. C, The median fluorescence intensity (MFI) is shown for Sca-1 staining with a PE-conjugated antibody post-fixation with PFA and permeabilization with the indicated agents. MFIs are from the LIN-Kit+Sca-1+ gate. The MFI from PFA-fixed/acetone-permeabilized cells was reduced compared to PFA-fixed cells that were not permeabilized in the LIN-Kit+Sca-1+ gate. Data are from a representative experiment.
(0.99 MB TIF)

Figure S2 Flt3 and CD48 staining. A, Cells were left untreated or fixed and permeabilized (fix/perm) with PFA and acetone, respectively, and stained with FITC-conjugated Sca-1, APC-conjugated c-Kit and PE-conjugated Flt3 antibodies. The percentage of cells staining positive in each population subset for Flt3 is indicated from one representative experiment. Notice the fluorescence intensity of Flt3 post-fix/perm is reduced although there is retention in percent positive cells relative to untreated samples. B, Cells were treated as in (A) and stained with Pacific Blue-conjugated CD48 and APC-conjugated c-Kit antibodies. The percentage of cells staining positive in each population subset for CD48 is indicated from one representative experiment
(7.31 MB TIF)

Figure S3 Quantification of signaling changes in response to Scf or Thpo in HSC/HPCs. The fold change (Δ) in MFI was calculated by dividing the MFI of stimulated/drug-treated cells with that of unstimulated cells. For each agonist, values are normalized to unstimulated cells in individual cell subsets (LSK values are relative to unstimulated LSK; LK values are relative to unstimulated LK). Bar graphs represent the means from 2 independent experiments. Error bars indicate the SD. Abbreviations are as in Figure 2.
(5.86 MB TIF)

Figure S4 No change in extracellular surface marker levels in agonist/drug-treated HSC/HPC. Cells were treated as in Figure 2, and the percentages of LSK, LK, and LDN cells (as in Figure 1) were assessed. Data are representative of 2–3 independent experiments, and percentage of the parental gate from 1 experiment is shown.
(7.63 MB TIF)

Figure S5 Quantification of changes in MFI of phosphoprotein epitopes in response to other agonists. The fold change (Δ) in MFI was calculated by dividing the MFI of stimulated/drug-treated cells with that of unstimulated cells as in Figure S3. Bar graphs represent the means from 2 independent experiments. Error bars indicate the SD. Abbreviations are as in Figure 2.
(5.35 MB TIF)

Figure S6 Responses of CD34/phosphoprotein subsets to Scf or Thpo stimulation. Cells were treated as in Figure 2, and gated for the indicated cell surface and intracellular markers with or without stimulation by the indicated agonists. Results are representative of 2–3 independent experiments, with the percentage of the parental gates from 1 experiment indicated
(6.79 MB TIF)

Figure S7 Responses of CD34/phosphoprotein subsets to other agonists. Cells were treated as in Figure 2, and gated for the indicated cell surface and intracellular markers with or without stimulation by the indicated agonists. Results are representative of 2 independent experiments, with the percentage of the parental gates from 1 experiment indicated. NC, No Change.
(7.09 MB TIF)
