# Supplementary material for: Flow-Cytometric Phosphoprotein Analysis Reveals Agonist and Temporal Differences in Responses of Murine Hematopoietic Stem/Progenitor Cells
Source: PLoS One. 2008 Nov 20;3(11):e3776. doi: 10.1371/journal.pone.0003776 (PMC2582484; doi:10.1371/journal.pone.0003776)
Supplement: Materials and Methods S1 — (0.03 MB DOC) [file pone.0003776.s001.doc]

**Flow-Cytometric Phosphoprotein Analysis** **Reveals Agonist and Temporal Differences in Responses of Murine Hematopoietic Stem/Progenitor Cells**

Demetrios Kalaitzidis and Benjamin G. Neel

**Supporting Information: Materials and Methods S1**

**Antibodies**

Antibodies against Sca-1 (FITC-conjugate, E13-161.7, BD), used at 5 ng/μl for untreated and 0.65 ng/μl for fix/perm cells, Flt3/Flk2 (PE-conjugate, A2F10.1, BD), used at 4 ng/μl for untreated and 2 ng/μl for fix/perm cells, and CD48 (Pacific-Blue conjugate, HM48-1, BioLegend), used at 5 ng/μl, were used to generate some of the supporting data.
