# Supplementary figures and images for: Flow-Cytometric Phosphoprotein Analysis Reveals Agonist and Temporal Differences in Responses of Murine Hematopoietic Stem/Progenitor Cells
Source: PLoS One. 2008 Nov 20;3(11):e3776. doi: 10.1371/journal.pone.0003776 (PMC2582484; doi:10.1371/journal.pone.0003776)

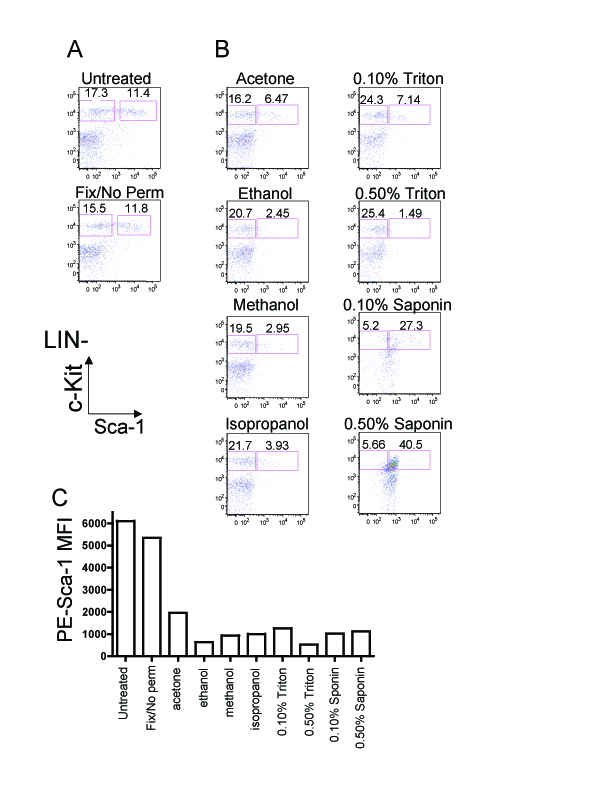

Supplement: Figure S1 — Effect of fixation/permeabilization conditions on Sca-1 staining. A, Cells were either left untreated or fixed with PFA without permeabilization, and subsequently stained for Kit and Sca-1. B, Cells were treated as in (A), and permeabilized with the indicated agents post-fixation. A mimimum of 1,400 FSC/SSC-gated events was collected for each treatment. Saponin was included in the permeabilization and wash/staining buffer when used. C, The median fluorescence intensity (MFI) is shown for Sca-1 staining with a PE-conjugated antibody post-fixation with PFA and permeabilization with the indicated agents. MFIs are from the LIN-Kit+Sca-1+ gate. The MFI from PFA-fixed/acetone-permeabilized cells was reduced compared to PFA-fixed cells that were not permeabilized in the LIN-Kit+Sca-1+ gate. Data are from a representative experiment. (0.99 MB TIF) [file pone.0003776.s002.tif]

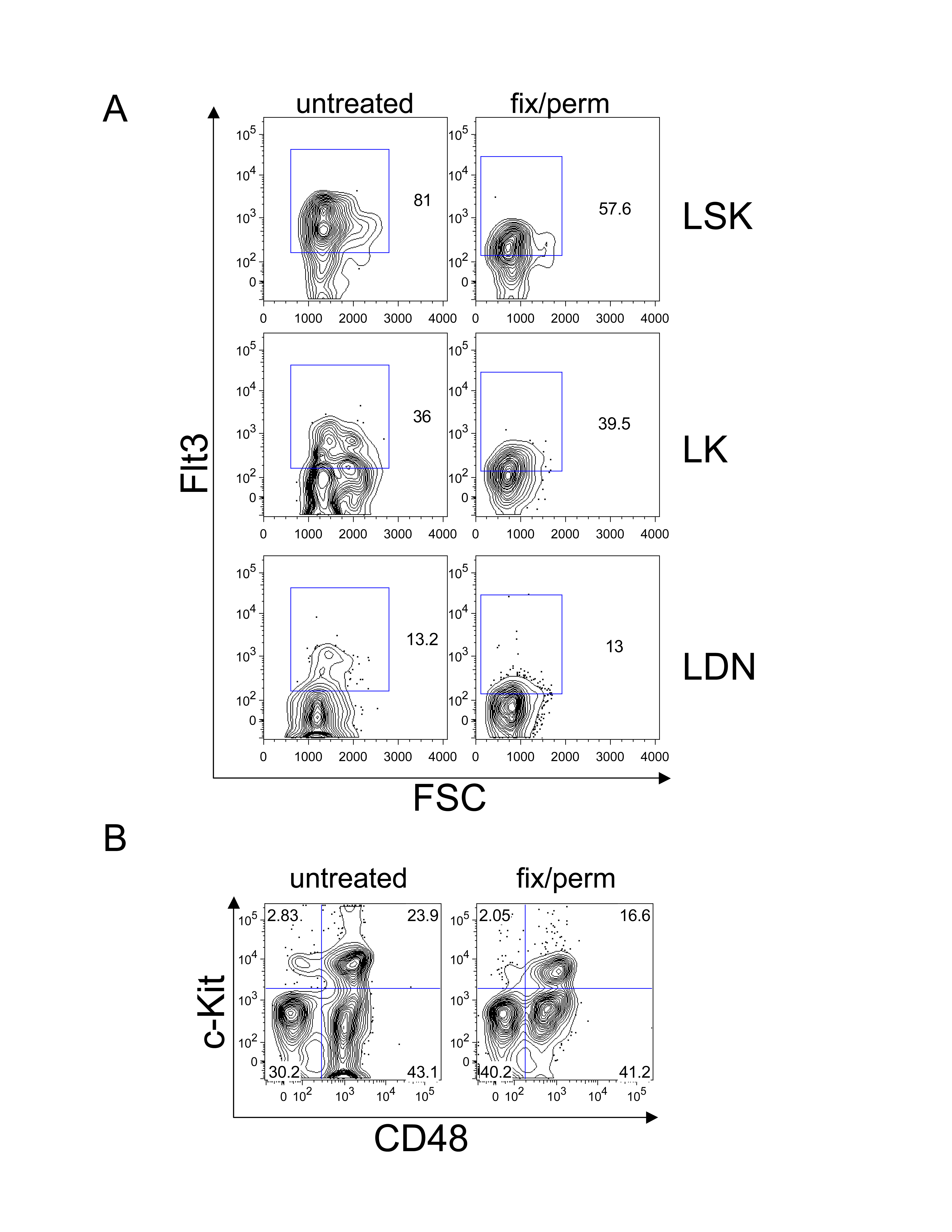

Supplement: Figure S2 — Flt3 and CD48 staining. A, Cells were left untreated or fixed and permeabilized (fix/perm) with PFA and acetone, respectively, and stained with FITC-conjugated Sca-1, APC-conjugated c-Kit and PE-conjugated Flt3 antibodies. The percentage of cells staining positive in each population subset for Flt3 is indicated from one representative experiment. Notice the fluorescence intensity of Flt3 post-fix/perm is reduced although there is retention in percent positive cells relative to untreated samples. B, Cells were treated as in (A) and stained with Pacific Blue-conjugated CD48 and APC-conjugated c-Kit antibodies. The percentage of cells staining positive in each population subset for CD48 is indicated from one representative experiment (7.31 MB TIF) [file pone.0003776.s003.tif]

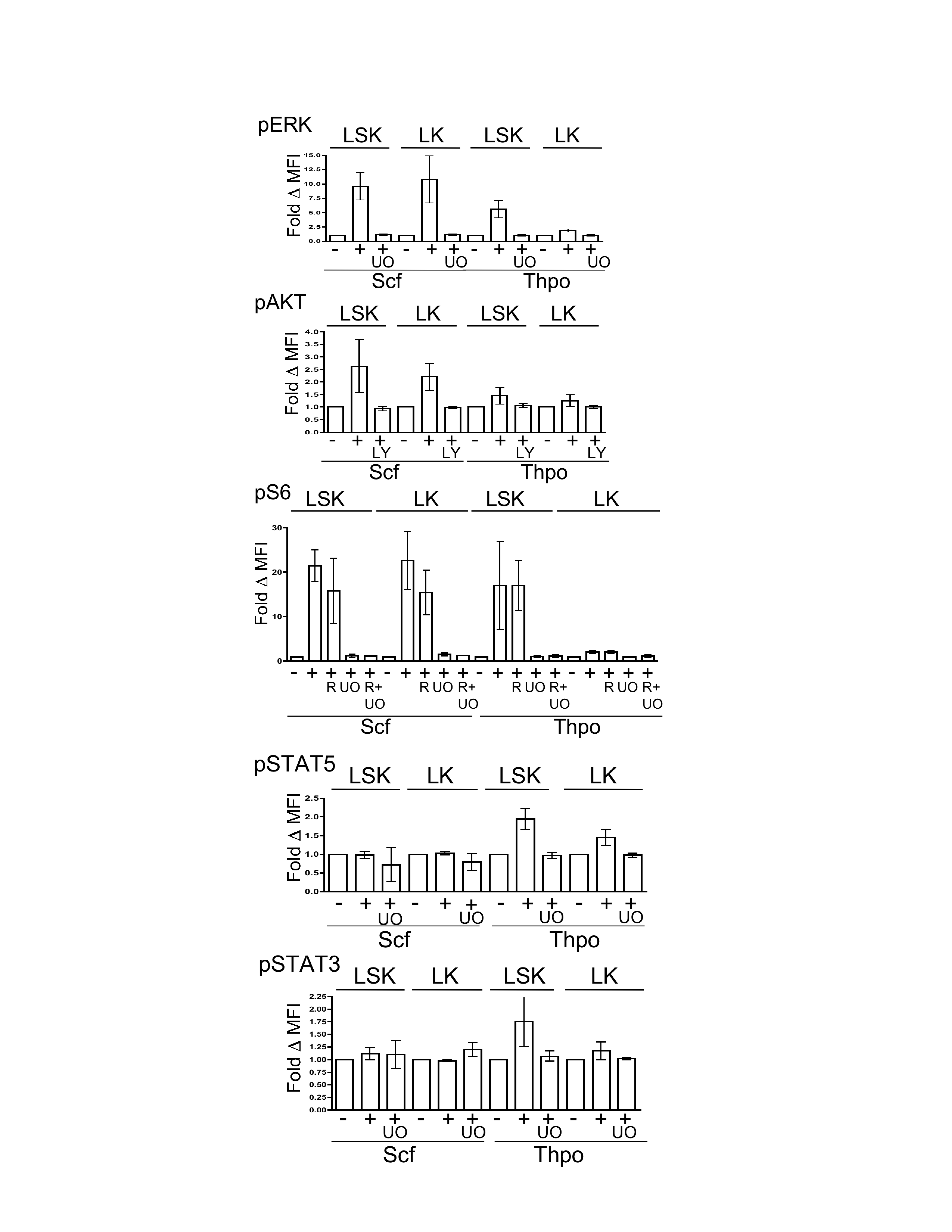

Supplement: Figure S3 — Quantification of signaling changes in response to Scf or Thpo in HSC/HPCs. The fold change (Δ) in MFI was calculated by dividing the MFI of stimulated/drug-treated cells with that of unstimulated cells. For each agonist, values are normalized to unstimulated cells in individual cell subsets (LSK values are relative to unstimulated LSK; LK values are relative to unstimulated LK). Bar graphs represent the means from 2 independent experiments. Error bars indicate the SD. Abbreviations are as in Figure 2. (5.86 MB TIF) [file pone.0003776.s004.tif]

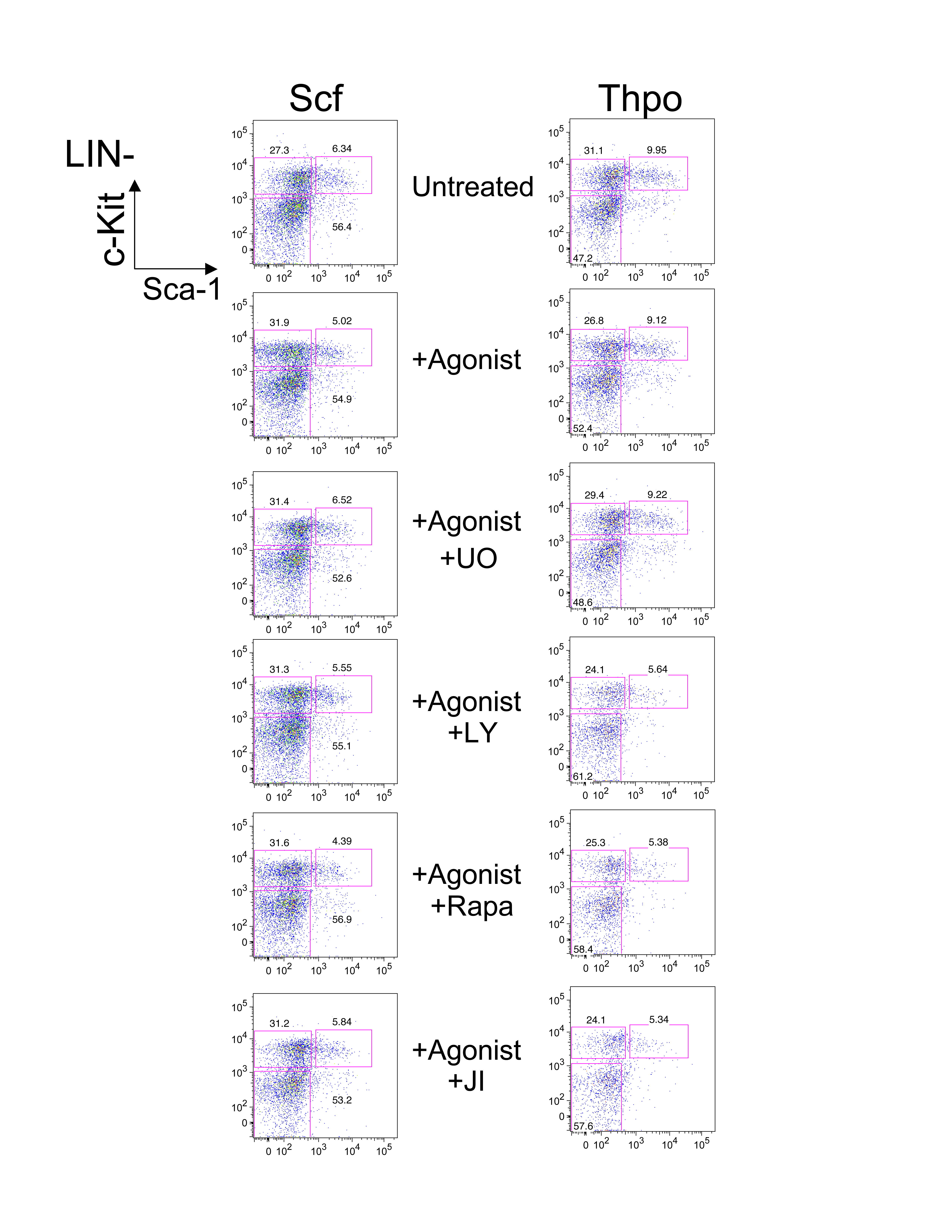

Supplement: Figure S4 — No change in extracellular surface marker levels in agonist/drug-treated HSC/HPC. Cells were treated as in Figure 2, and the percentages of LSK, LK, and LDN cells (as in Figure 1) were assessed. Data are representative of 2–3 independent experiments, and percentage of the parental gate from 1 experiment is shown. (7.63 MB TIF) [file pone.0003776.s005.tif]

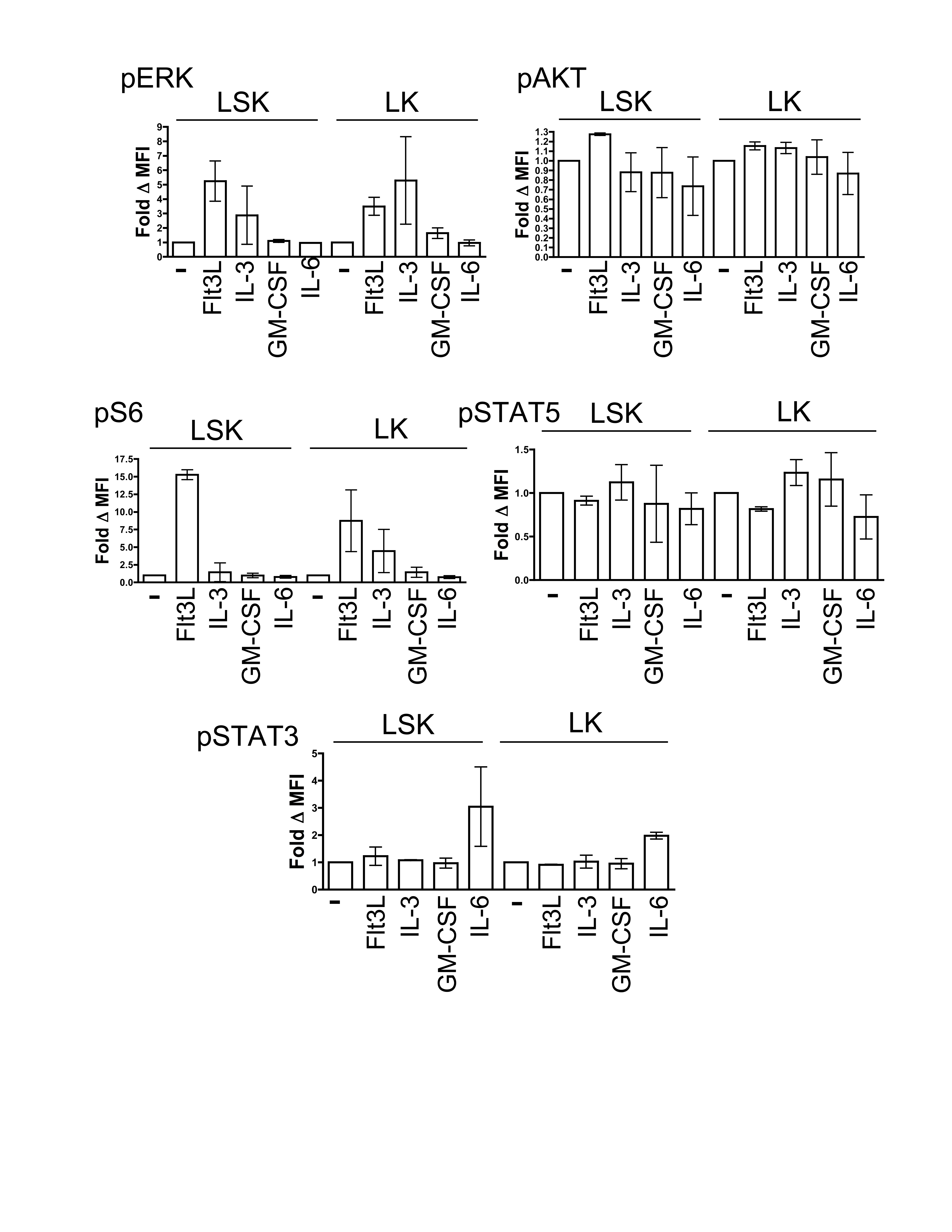

Supplement: Figure S5 — Quantification of changes in MFI of phosphoprotein epitopes in response to other agonists. The fold change (Δ) in MFI was calculated by dividing the MFI of stimulated/drug-treated cells with that of unstimulated cells as in Figure S3. Bar graphs represent the means from 2 independent experiments. Error bars indicate the SD. Abbreviations are as in Figure 2. (5.35 MB TIF) [file pone.0003776.s006.tif]

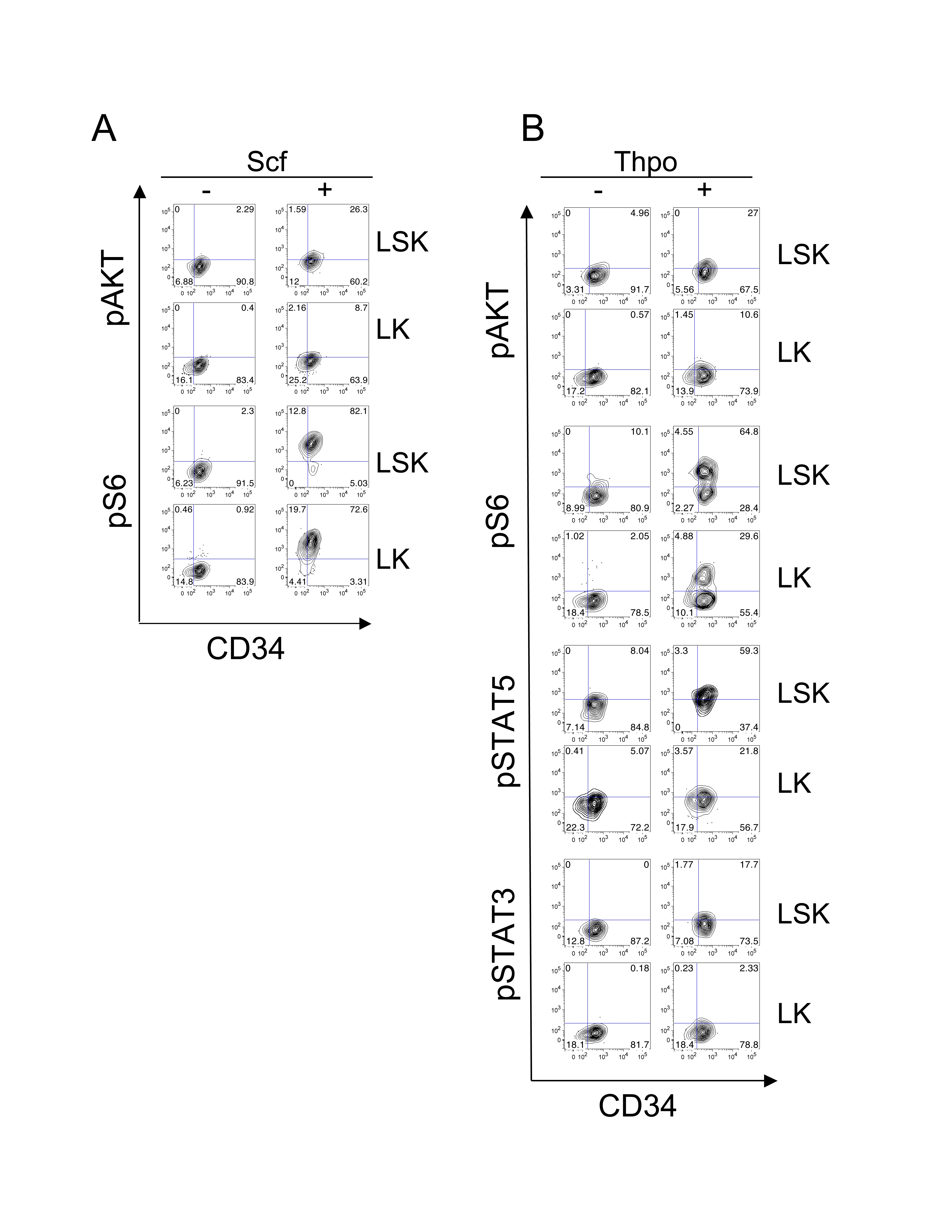

Supplement: Figure S6 — Responses of CD34/phosphoprotein subsets to Scf or Thpo stimulation. Cells were treated as in Figure 2, and gated for the indicated cell surface and intracellular markers with or without stimulation by the indicated agonists. Results are representative of 2–3 independent experiments, with the percentage of the parental gates from 1 experiment indicated (6.79 MB TIF) [file pone.0003776.s007.tif]

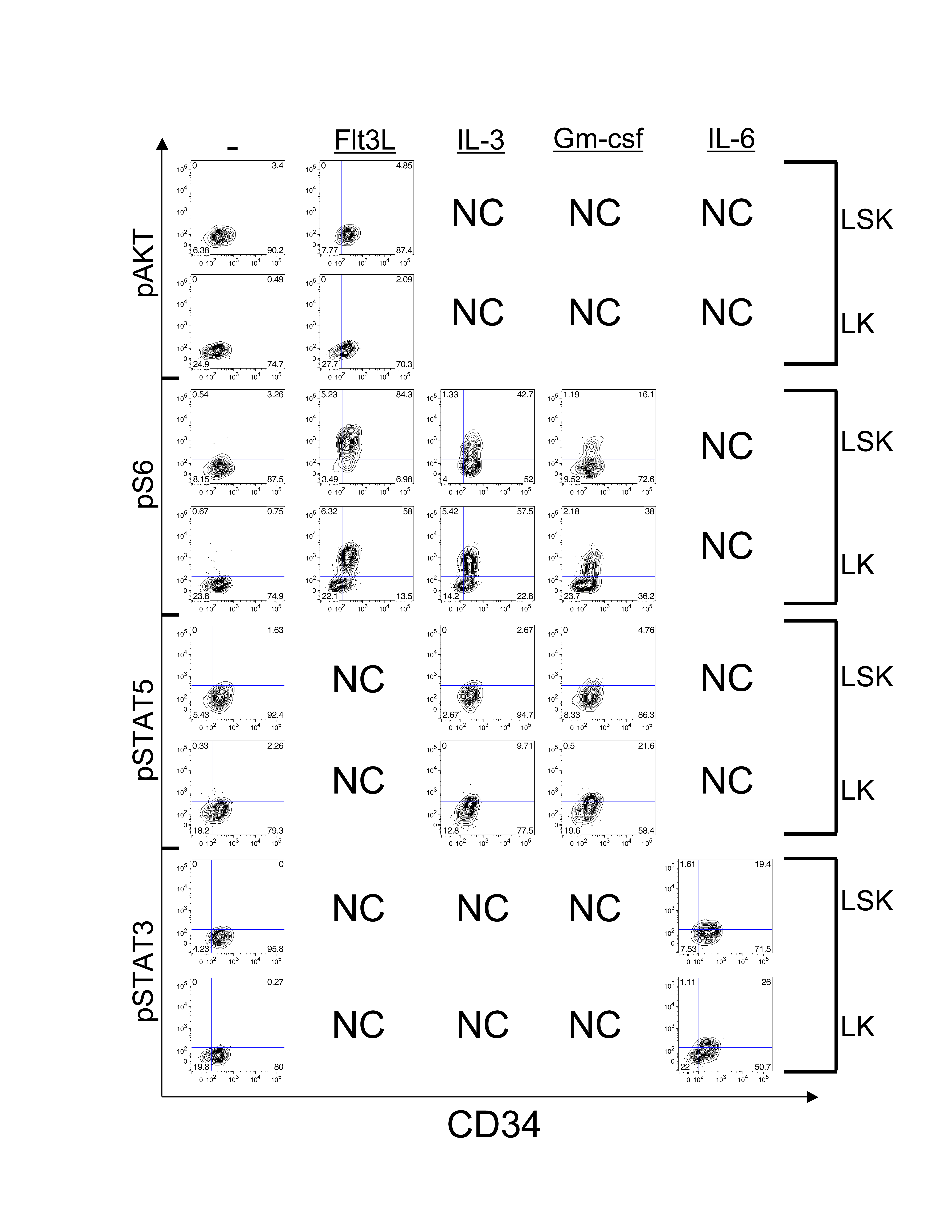

Supplement: Figure S7 — Responses of CD34/phosphoprotein subsets to other agonists. Cells were treated as in Figure 2, and gated for the indicated cell surface and intracellular markers with or without stimulation by the indicated agonists. Results are representative of 2 independent experiments, with the percentage of the parental gates from 1 experiment indicated. NC, No Change. (7.09 MB TIF) [file pone.0003776.s008.tif]
